# Citrate Regulates the *Saccharomyces cerevisiae* Mitochondrial GDP/GTP Carrier (Ggc1p) by Triggering Unidirectional Transport of GTP

**DOI:** 10.3390/jof8080795

**Published:** 2022-07-29

**Authors:** Roberta Seccia, Silvia De Santis, Maria A. Di Noia, Ferdinando Palmieri, Daniela V. Miniero, Raffaele Marmo, Eleonora Paradies, Antonella Santoro, Ciro L. Pierri, Luigi Palmieri, Carlo M. T. Marobbio, Angelo Vozza

**Affiliations:** 1 Department of Biosciences, Biotechnologies and Biopharmaceutics, University of Bari, 70125 Bari, Italy; roberta.seccia@uniba.it (R.S.); silvia.desantis@uniba.it (S.D.S.); maria.dinoia@uniba.it (M.A.D.N.); ferdpalmieri@gmail.com (F.P.); danielavaleria.miniero@uniba.it (D.V.M.); rafmarmo@gmail.com (R.M.); antonella.santoro@uniba.it (A.S.); ciro.pierri@uniba.it (C.L.P.); luigi.palmieri@uniba.it (L.P.); 2 CNR Institute of Biomembranes, Bioenergetics and Molecular Biotechnologies (IBIOM), 70125 Bari, Italy; e.paradies@ibiom.cnr.it; 3 Center of Excellence in Comparative Genomics, University of Bari, Via Orabona 4, 70125 Bari, Italy

**Keywords:** citrate, GDP/GTP carrier, GTP, Fe-S centers, membrane transport, mitochondria, mitochondrial carrier

## Abstract

The yeast mitochondrial transport of GTP and GDP is mediated by Ggc1p, a member of the mitochondrial carrier family. The physiological role of Ggc1p in *S. cerevisiae* is probably to transport GTP into mitochondria in exchange for GDP generated in the matrix. *ggc1*Δ cells exhibit lower levels of GTP and increased levels of GDP in mitochondria, are unable to grow on nonfermentable substrates and lose mtDNA. Because in yeast, succinyl-CoA ligase produces ATP instead of GTP, and the mitochondrial nucleoside diphosphate kinase is localized in the intermembrane space, Ggc1p is the only supplier of mitochondrial GTP required for the maturation of proteins containing Fe-S clusters, such as aconitase [4Fe-4S] and ferredoxin [2Fe-2S]. In this work, it was demonstrated that citrate is a regulator of purified and reconstituted Ggc1p by trans-activating unidirectional transport of GTP across the proteoliposomal membrane. It was also shown that the binding site of Ggc1p for citrate is different from the binding site for the substrate GTP. It is proposed that the citrate-induced GTP uniport (CIGU) mediated by Ggc1p is involved in the homeostasis of the guanine nucleotide pool in the mitochondrial matrix.

## 1. Introduction

GTP is required in mitochondria in several processes conserved from yeast to humans. Mitochondrial DNA replication, repair and transcription require GTP as well as the initiation, elongation and termination steps of mitochondrial protein synthesis [1]. GTPases attached to the inner or outer mitochondrial membrane are necessary for morphologic changes in the organelle in fusion or fission processes [2,3]. Furthermore, the presence of several GTPases of unknown function in the mitochondrial matrix sheds light on the additional roles of GTP. For instance, in yeast mitochondria, GTP is most likely required for a common step involved in the Fe-S cluster biogenesis of Fe-S cluster-containing proteins, such as aconitase [4Fe-4S] and ferredoxin [2Fe-2S] [4]. Despite the evolutionarily conserved GTP processes inside mitochondria, GTP synthesis and transport are differently compartmentalized in mammals and yeast. In mammals, the mitochondrial synthesis of GTP is catalyzed by two different enzymes: (i) the succinyl-CoA ligase in the Krebs cycle that converts succinyl-CoA to succinate with the production of GTP; (ii) the nucleoside diphosphate kinase, which catalyzes the transfer of a phosphate group from nucleoside triphosphates, mainly ATP, to nucleoside diphosphates [5]. In yeast mitochondria, GTP cannot be synthesized because succinyl-CoA ligase produces ATP [6], and the yeast nucleoside diphosphate kinase, encoded by a single nuclear gene (*YNK1*) [7], is localized in the cytosol and mitochondrial intermembrane space but not in the matrix [8]. The yeast GTP import from the cytosol into mitochondria was discovered by the identification and functional characterization of Yhm1p [9]. In fact, in 2004, Yhm1p [10] was identified as the GTP/GDP transporter, named Ggc1p (T.C. number: 2.A.29.21.1), which catalyzes the exchange of cytosolic GTP for matrix GDP across the inner mitochondrial membrane [9]. This mitochondrial protein belongs to the mitochondrial carrier (MC) protein superfamily [11], which is characterized by three tandem sequence repeats, each being approximately 100 amino acids in length and folded into two transmembrane α-helices joined by an extensive hydrophilic loop. Ggc1 knockout cells are unable to grow on nonfermentable substrates, exhibit lower levels of GTP and increased levels of GDP in their mitochondria, and lose their mtDNA [9]. Additional physiological roles of Ggc1p were discovered later: (i) transcriptome and proteome analysis of yeast cells treated for 6 h with rapamycin, the well-known TOR pathway inhibitor, identified Ggc1p as a new component of the rapamycin signaling pathway because, in the presence of rapamycin, the Ggc1p was stabilized and the growth fitness of the *ggc1*Δ strain was enhanced [12]; (ii) Ggc1p recruited Mec1 during the formation of the Snf1-Mec1-Atg1 module, which is important for maintaining mitochondrial respiration and energy deprivation-activated autophagy during glucose starvation [13]; (iii) Ggc1p is involved in mitochondrial iron homeostasis, given that *ggc1*Δ strain accumulates iron inside mitochondria [14]. Interestingly, the iron phenotypic defects were reported to be rescued by the over-expression of the human nucleoside diphosphate kinase Nm23-H4 targeted to the mitochondrial matrix, where it converts GDP to GTP using ATP as phosphate donor [4]. Despite some evidence about the link between mitochondrial iron homeostasis and the TOR pathway in yeast and mammals [15,16,17], the relationship between mitochondrial iron, guanine nucleotides content and TOR pathway remains to be elucidated. In the present study, it was demonstrated that citrate regulates Ggc1p by trans-activating the uniport of GTP into the mitochondria, suggesting a role of citrate in the homeostasis of the guanine nucleotide pool in the mitochondrial matrix. To the best of our knowledge, this is the first time that a mitochondrial carrier has been shown to be regulated by a metabolite that is not transported by the same carrier.

## 2. Materials and Methods

### 2.1. Bacterial Expression and Purification of Wild-Type and Mutant Ggc1p

The forward and reverse oligonucleotide primers corresponded to the extremities of the coding sequence of *GGC1,* followed by the stop codon and the additional *BamHI* and *HindIII* restriction sites at 5’ and 3’ ends, respectively. The single mutants of Ggc1p were obtained by replacing the native R53, K154, R156 and R251 amino acids with alanine in the template of the *GGC1* gene. The mutations were introduced in the wild-type *GGC1* gene by the overlap extension PCR method using oligonucleotides carrying the appropriate mutation in their sequences, as previously reported [18,19,20,21,22]. The PCR products were cloned into the pMW7 expression vector and transformed into *E. coli* DH5α cells. Transformants, which were selected on LB plates containing ampicillin (100 μg/mL), were screened by direct colony PCR and by restriction digestion of the purified plasmids DNA. All constructs were verified by DNA sequencing. The overexpression of *GGC1* constructs resulted in the production of inclusion bodies in the cytosol of *E. coli* BL21(DE3), and it was accomplished as previously described [9,23]. Quantitative evaluation of recombinant proteins was estimated from Coomassie Blue-stained SDS-PAGE gels of Coomassie-stained protein bands, using the Chemidoc imaging system equipped with Quantity One software (Bio-Rad, software version number 4.6.9) [24]. Inclusion bodies were purified on a sucrose density gradient and washed at 4 °C, firstly with TE buffer (10 mM Tris/HCl, 1 mM ethylenediaminetetraacetic acid (EDTA), pH 8.0), then twice with a buffer containing Triton X-114 (1.8%, *w*/*v*) and 10 mM PIPES (pH 6.5), and finally with PIPES 10 mM (pH 6.5). The identity of the recombinant protein was assayed by immunoblotting [25,26] using a rabbit polyclonal antibody against recombinant Ggc1p.

### 2.2. Yeast Strains, Media and Growth Conditions

The *S. cerevisiae* wild-type BY4741 (*MATa*; *his3Δ1*; *leu2Δ0*; *met15Δ0*; *ura3Δ0*) and the isogenic *ggc1*Δ yeast strains were provided by the EUROFAN resource center EUROSCARF (Frankfurt, Germany). In the *ggc1*Δ mutant, the *GGC1* (YDL198c) locus was replaced by kanMX4 (Frankfurt, Germany). The wild-type and knockout strain were grown in rich YP medium containing 2% galactose [27]. Mitochondria were isolated by standard procedures from cells grown until the early exponential phase (optical density between 1.0 and 1.5) was reached [28].

### 2.3. Reconstitution into Liposomes and Transport Assays

The recombinant proteins solubilized in 1.8% (*w*/*v*) sarkosyl (Sigma-Aldrich, St. Louis, MO, USA) were reconstituted into liposomes in the presence or absence of substrates [9,29,30,31]. For transport experiments of liposomes reconstituted with yeast mitochondrial extracts, 30 μg of isolated mitochondria were solubilized (0.5 mg/mL) in a buffer containing 3% TX-114, 1 mM EDTA and 10 mM PIPES, pH 7.0 for 45 min on ice, and reconstituted in liposomes [32,33]. The reconstitution mixture contained the recombinant Ggc1p or mitochondrial extracts, including Ggc1p protein, 1.2% (*w*/*v*) Triton X114, 1.3% (*w*/*v*) egg yolk phosphatidylcholine (Sigma-Aldrich) sonicated liposomes, 0.4 mg/mL cardiolipin (Sigma-Aldrich), 5 mM unlabeled substrate (except where otherwise indicated), 10 mM PIPES at pH 7.0, and water to a final volume of 700 µL. The mixture was vortexed and recycled for 13 times through the same Amberlite column (Bio-Rad) [9,34]. The external unlabeled substrate was removed from proteoliposomes on a Sephadex G75 column pre-equilibrated with 50 mM NaCl and 10 mM PIPES (pH 7.0). Transport at 25 °C was started by adding the labeled substrate, at the indicated concentrations, to unlabeled substrate-loaded proteoliposomes or empty proteoliposomes [9,35] and terminated, at the desired time point, by the addition of 30 mM pyridoxal 5′-phosphate (PLP) and 15 mM bathophenanthroline (BAT). In control samples, these inhibitors were added together with the labeled substrate at time 0, according to the inhibitor stop method [9,36,37]. Finally, the external substrate was removed by exclusion chromatography, and the radioactivity in proteoliposomes was measured. Experimental values were corrected by subtracting control values. The initial transport rate was calculated from the radioactivity taken up by proteoliposomes after 20 s (in the initial linear range of substrate uptake). For efflux measurements, proteoliposomes containing 0.2 mM GTP were labeled with 20 µM [8-^3^H GTP] by carrier-mediated exchange equilibration [38]. After 40 min, the external substrate was removed by exclusion chromatography using Sephadex G-75 columns pre-equilibrated with 50 mM NaCl and 10 mM PIPES, pH 7.0. Efflux was started by adding 5 mM GTP, 5 mM citrate or 2.5 mM NaCl. In all cases, the transport was terminated by adding 30 mM PLP and 15 mM BAT [9,39].

### 2.4. Comparative Modeling and Docking Investigations

Computational approaches [40,41] were employed to investigate the mechanism of citrate-mediated GTP uptake into proteoliposomes. At first, an inter-repeat sequence alignment [34,40,42] between the yeast Ggc1p and the crystallized bovine ADP/ATP carrier 1 sequences (BtAAC1, protein data bank accession code: 1okc [43]) was obtained by using ClustalW for highlighting residues between the first and the second part of MC sequence motif PX[D/E]XX[K/R]X[R/K]…..EGXXXXAr[K/R]G [44,45] is putatively involved in the citrate-mediated GTP uptake. Then, a 3D comparative model of Ggc1p in cytosolic conformation was obtained by using SwissPDBViewer (SPDBV, http://spdbv.vital-it.ch/, accessed on 15 May 2022) [46] and the structure of the BtAAC1 (1okc.pdb), crystallized in complex with its powerful inhibitor carboxyatractyloside (CATR) as a protein template for the modeling session [40,47,48]. The percentage of identical residues between Ggc1p and BtAAC1 is rather low (22%). However, the residues of the sequence motif of the mitochondrial carrier family members were employed as anchor points for preparing the sequence structure pairwise alignment used to generate the 3D comparative model, as previously performed for other mitochondrial carriers. During the modeling process, the Cα backbone of the Ggc1p target was restrained to the backbone of the template structure, and residue side chains were added by the software, as previously described. The generated 3D comparative model was energetically minimized by using the SPDBV energy minimization tool. The single amino acid replacement of residues R53, K154, R156 and R251 with an alanine residue was obtained from *in silico* mutagenesis by using PyMOL [34,40,42,45,47] for investigating the role of the four basic residues in antiport activity and CIGU. The structural properties of the obtained wild-type Ggc1p comparative model and those of the above-cited four Ggc1p mutant (R53A, K154A, R156A and R251A) models with the best energy function were evaluated by using the biochemical/computational tools of the WHAT IF Web server [34,40,42,45,47]. Docking analysis was performed by using Autodock 1.5.6 according to [47]. More in detail, the employed grid box consisted of 30, 30 and 36 grid points along the x-, y- and z-axis, respectively. The size of the grid box was chosen in order to contain all the residues within 4 Å from residues K154 and R156. The grid box spacing (i.e., the space between two adjacent grid points) was 0.275 Å, and the xyz coordinates of the grid center were 34.339 (x), 15.769 (y), and 8.646 (z). For the docking protocol, the maximum number of energy evaluations was set to 2,500,000, the maximum number of generations to 27,000, rmstol to 2.0 Å, and the number of the generated lowest free energy binding poses to 20. The lowest predicted free energy binding pose was used to investigate the binding interactions with the residues of the putative regulatory binding region.

## 3. Results

### 3.1. Bacterial Expression of Ggc1p

**Figure 1:**
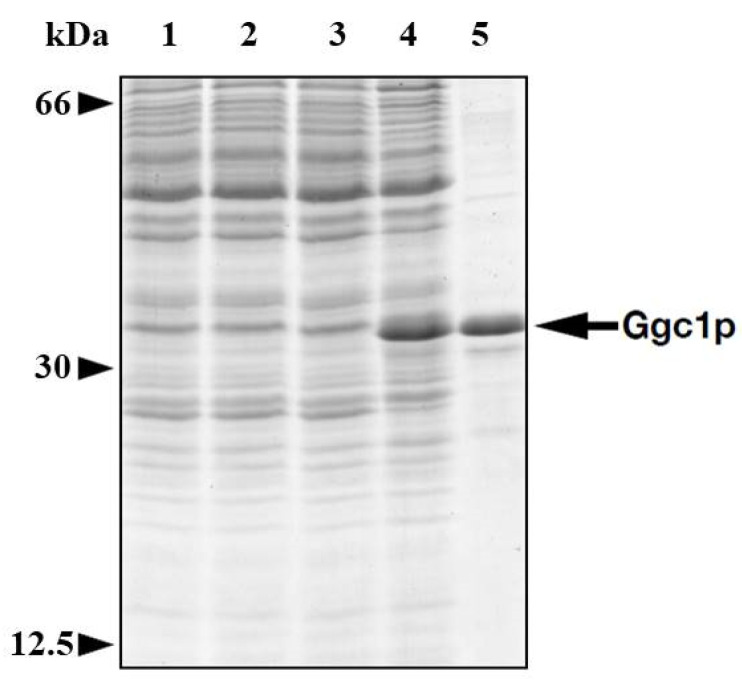
Bacterial overexpression and purification of Ggc1p. Proteins were separated by SDS–PAGE and stained with Coomassie blue dye. Markers: 66 kDa, bovine serum albumin; 30 kDa, carbonic anhydrase; 12.5 kDa cytochrome c; lanes 1–4, *Escherichia coli* BL21(DE3) containing the expression vector with the coding sequence of *GGC1* (lanes 2 and 4), or the empty vector (lanes 1 and 3). Lane 5, purified Ggc1p (4 µg) originating from bacteria shown in lane 4. Samples were taken at the time of induction (lanes 1 and 2) and 5 h later (lanes 3 and 4). The same number of bacterial cells was analyzed in each sample (30 μg of cellular proteins).

Ggc1p was expressed at high levels in *E. coli* BL21(DE3) (Figure 1, lane 4). It accumulated in the bacterial cytoplasm as inclusion bodies and was purified by centrifugation and washing (Figure 1, lane 5). The apparent molecular weight of the purified recombinant protein was 33.5 kDa, in agreement with the calculated value with initiator methionine (33,215 Da). The identity of the purified protein was confirmed by immunoblotting (not shown). Approximately 30–40 mg of purified protein were obtained per liter of culture. The protein was not detected in bacteria transformed with empty vector and harvested after induction (Figure 1, lane 3) nor in cells harvested immediately before induction of expression (Figure 1, lane 2).

### 3.2. Functional Characterization of Recombinant Ggc1p

**Figure 2:**
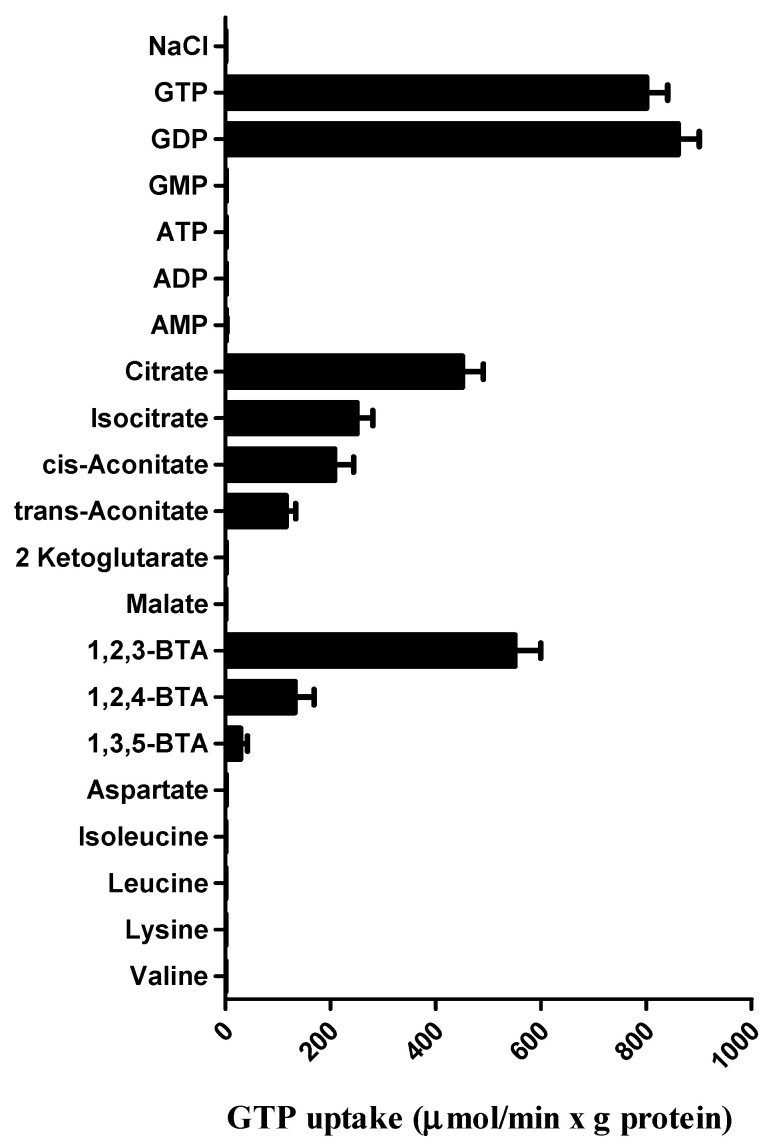
Dependence of Ggc1p transport activity on internal substrate. Proteoliposomes were preloaded internally with the reported substrates (5 mM). Transport was started by adding 1 μM [8-^3^H] GTP to proteoliposomes reconstituted with Ggc1p and stopped after 20 s.

Given that the previous results were focused on nucleotide transport catalyzed by Ggc1p [9], we aimed to examine the substrate specificity in greater detail by measuring the uptake of [8-^3^H]GTP into proteoliposomes that had been preloaded with various potential substrates, using external and internal substrate concentrations of 1 µM and 5 mM, respectively (Figure 2). High rates of [8-^3^H]GTP uptake into proteoliposomes were observed with internal GDP and GTP, as previously reported [9]. Surprisingly, GTP uptake was also observed with proteoliposomes containing citrate, isocitrate, cis-aconitate and 1,2,3-benzenetricarboxylic acid (1,2,3-BTA), which is the known specific impermeable citrate carrier inhibitor [49]. Some transport activity was also observed in the presence of internal trans-aconitate, 1,2,4-BTA and 1,3,5-BTA. All transport activities were strongly inhibited by a mixture of 30 mM PLP and 15 mM BAT, inhibitors of many mitochondrial carriers [9,10,11,12,13,14,15,16,17,18,19,20,21,22,23,24,25,26,27,28,29,30,31,32,33,34,35,36,37,38,39]. Recombinant and reconstituted Ggc1p did not catalyze heteroexchange of [8-^3^H]GTP for GMP, ATP, ADP, AMP, malate, 2-ketoglutarate, aspartate, isoleucine, leucine, lysine and valine (Figure 2). No [8-^3^H]GTP uptake activity was observed with Ggc1p that had been boiled before incorporation into liposomes nor by reconstitution of sarkosyl-solubilized material from bacterial cells either lacking the expression vector for Ggc1p or harvested immediately before the induction of expression (not shown).

### 3.3. Kinetic Characteristics of Recombinant Ggc1p

**Figure 3:**
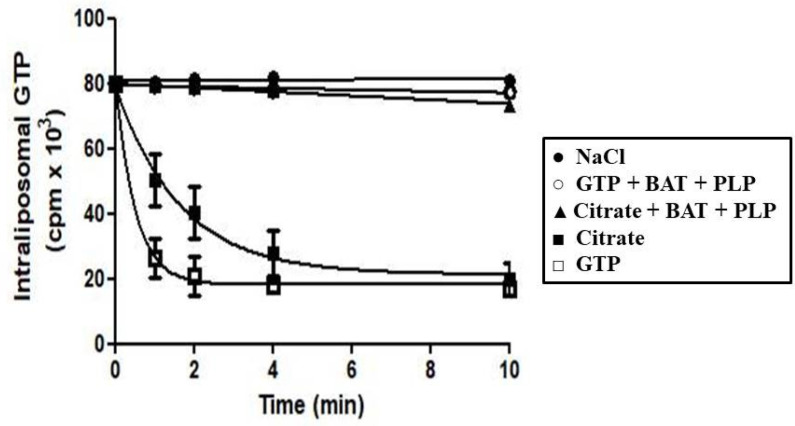
Efflux of GTP from proteoliposomes reconstituted with Ggc1p. The proteoliposomes were preloaded with 0.2 mM GTP, and then the internal substrate pool was made radioactive by carrier-mediated exchange equilibration. After 40 min, residual external radioactivity was removed by passing the proteoliposomes through a column of Sephadex G-75. The assay was started by adding 5 mM NaCl (●), 5 mM GTP (□), 5 mM citrate (■), 15 mM BAT, 30 mM PLP and 5 mM GTP (○) or 15 mM BAT, 30 mM PLP and 5 mM citrate (▲).

In Figure 3, the efflux of [8-^3^H]GTP from prelabeled active proteoliposomes was investigated. In the absence of an external substrate, no efflux was observed even after incubation for 10 min. By contrast, upon the addition of external GTP or citrate, an extensive efflux of radioactivity occurred, and this efflux was prevented completely by the presence of the PLP and BAT inhibitors (Figure 3). These results indicate that, at least under the experimental conditions used, externally added citrate induces the efflux of GTP from proteoliposomes via Ggc1p and raises the question of whether citrate, although very different from a nucleotide, is a substrate of Ggc1p.

**Figure 4:**
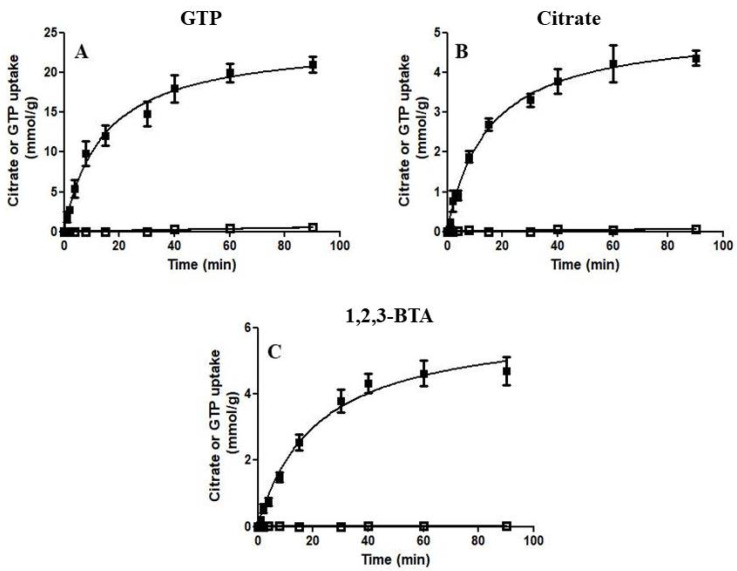
Time courses of [8-^3^H] GTP (■) and [1,5-^14^C] citrate (□) uptake in proteoliposomes reconstituted with the recombinant Ggc1p. 1 mM [8-^3^H] GTP or 1 mM of [1,5-^14^C] citrate was added to proteoliposomes containing internal 5 mM GTP (panel (**A**)), 5 mM citrate (panel (**B**)) or 5 mM 1,2,3-BTA (panel (**C**)).

**Figure 5:**
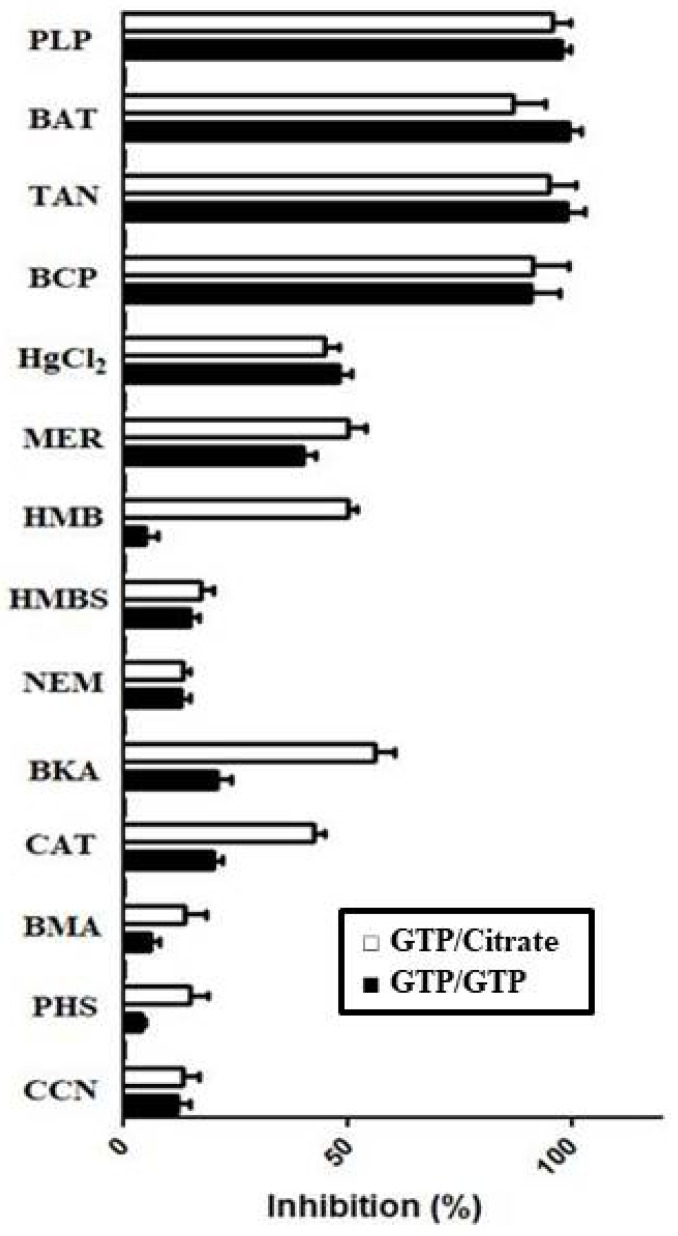
Effect of inhibitors on GTP/GTP exchange and citrate-induced GTP uptake by proteoliposomes reconstituted with Ggc1p. Proteoliposomes were preloaded internally with 5 mM GTP (black columns) or 5 mM citrate (white columns). Transport was started by adding 1 μM [8-^3^H] GTP and terminated after 20 s. All inhibitors were added together with [8-^3^H] GTP, but thiol reagents and α-cyanocinnamate were added 2 min before the labeled substrate. The final concentrations of the inhibitors were 0.05% (TAN, tannic acid), 0.1 mM (HgCl_2_, mercury chloride; HMB, p-hydroxymercuribenzoate; HMBS, p-hydroxymercuribenzene sulfonate; BKA, bongkrekic acid and CAT, carboxyatractyloside), 0.3 mM (BCP, bromocresol purple), 1 mM (CCN, α-cyanocinnamate) and 2 mM (PLP, pyridoxal 5′-phosphate; BAT, bathophenanthroline; NEM, N-ethylmaleimide; BMA, butylmalonate; PHS, phenylsuccinate).

In Figure 4, the transport kinetics are compared for the uptake of 1 mM [8-^3^H]GTP or 1 mM [^14^C]citrate into proteoliposomes in the presence of internal 5 mM GTP (Figure 4A), 5 mM citrate (Figure 4B) or 5 mM 1,2,3-BTA (Figure 4C). The results demonstrate that Ggc1p catalyzes [8-^3^H]GTP uptake when GTP, citrate or 1,2,3-BTA are present inside the proteoliposomes (Figure 4A–C). By contrast, no [^14^C]citrate uptake was observed in all these conditions (Figure 4A–C), providing clear evidence that citrate, and most likely 1,2,3-BTA, are not transported by Ggc1p.

Interestingly, a different inhibitor sensitivity was shown by Ggc1p when the proteoliposomes were preloaded internally with 5 mM citrate, compared with the results found in our previous study, where proteoliposomes were preloaded with 5 mM GTP [9]. As shown in Figure 5, both antiport (GTP/GTP exchange) and CIGU catalyzed by reconstituted Ggc1p were strongly inhibited by PLP and BAT (inhibitors of many mitochondrial carriers), tannic acid and bromocresol purple (inhibitors of the mitochondrial glutamate carriers). Both transport activities were only partially inhibited by sulfhydryl reagents such as mercury chloride and mersalyl. Unlike the exchange reaction, CIGU was partially inhibited by *p*-hydroxymercuribenzoate, carboxyatractyloside and bongkrekate. The latter two compounds are powerful inhibitors of the ADP/ATP carrier at much lower concentrations than those used in this study. A weak inhibitory effect was observed with *p*-hydroxymercuribenzene sulfonate, *N*-ethylmaleimide, butylmalonate, phenylsuccinate, and α-cyano-4-hydroxycinnamate (inhibitors of other mitochondrial carriers, Figure 5) [9].

**Figure 6:**
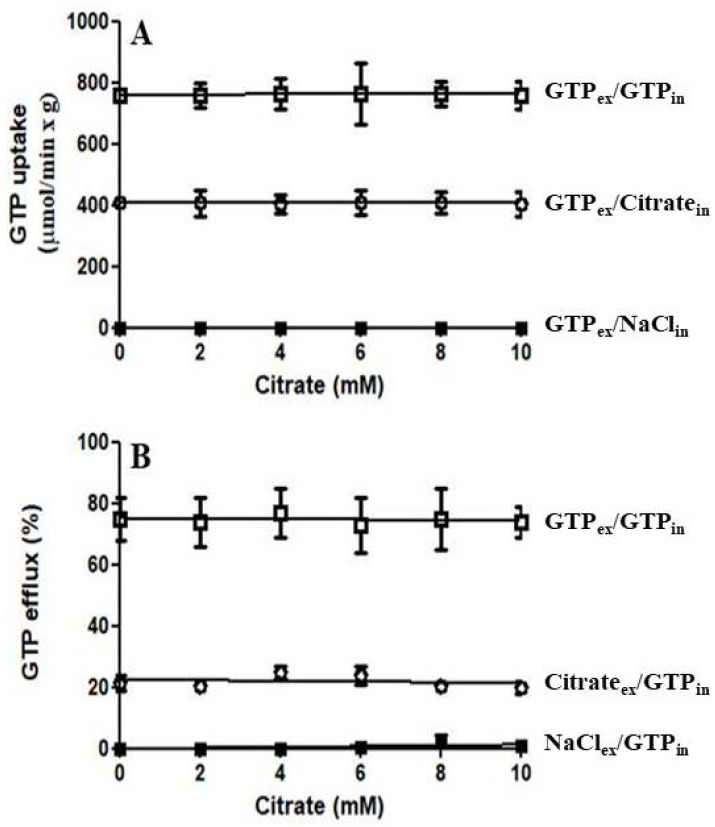
(**A**) The rate of GTP uptake into proteoliposomes reconstituted with Ggc1p is independent of external citrate. Transport was started by adding 1 μM [8-^3^H] GTP and citrate (0–10 mM) to proteoliposomes containing 5 mM citrate (○), 5 mM GTP (□) or 5 mM NaCl (■) and stopped after 20 s; (**B**) The rate of GTP efflux from proteoliposomes reconstituted with Ggc1p is independent of internal citrate. Proteoliposomes were reconstituted in the presence of 0.2 mM GTP and citrate (0–10 mM). The internal GTP pool was labeled by carrier-mediated exchange equilibration. Then the proteoliposomes were passed through Sephadex G-75 columns pre-equilibrated with 50 mM NaCl and 10 mM PIPES pH 7.0. The efflux of [8-^3^H] GTP was started by adding 1 mM citrate (○), 1 mM GTP (□) or 0.5 mM NaCl (■) and stopped after 20 s.

As shown in Figure 6A, the initial rate of [8-^3^H]GTP uptake into proteoliposomes preloaded with citrate (CIGU) or GTP (GTP/GTP homoexchange) does not depend on the external concentration of citrate (0–10 mM). Similarly, the efflux of [8-^3^H]GTP from prelabeled proteoliposomes induced by external GTP (homoexchange) or citrate (CIGU) does not depend on the intraliposomal concentration of citrate (Figure 6B). Therefore, these results suggest that the Ggc1p binding site for citrate is different from that for the substrate GTP.

**Figure 7:**
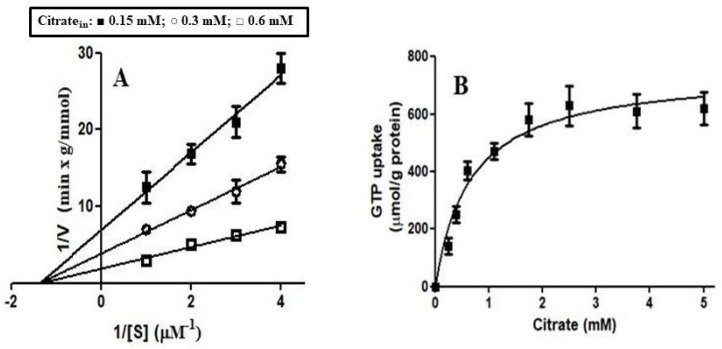
Dependence of the rate of CIGU in proteoliposomes reconstituted Ggc1p on the external GTP concentration and intraliposomal citrate concentration. (**A**) [8-^3^H] GTP was added at the concentrations indicated to proteoliposomes containing 0.15 (■), 0.3 (○) and 0.6 mM (□) citrate. (**B**) An amount of 10 μM [8-^3^H] GTP was added to proteoliposomes containing citrate at the concentrations indicated.

In order to obtain kinetic information on the citrate-induced [8-^3^H]GTP uptake into proteoliposomes reconstituted with Ggc1p, the dependence of the GTP uptake rate on internal citrate concentration was investigated by changing the concentration of externally added [8-^3^H]GTP (0.25 μM–1 μM) at three internal concentrations of citrate (0.15, 0.4 and 0.6 mM). As shown in Figure 7A, the *K*_m_ value for GTP (0.72 μM) remained constant independently from the citrate concentration. In contrast, the *V*_max_ increased on raising the concentration of citrate. Furthermore, CIGU shows saturation kinetics with an apparent activation constant (*K*_att_) equal to 0.51 mM for citrate (Figure 7B).

**Figure 8:**
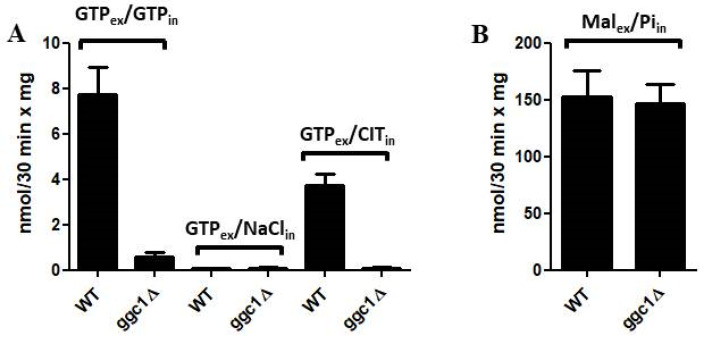
GTP/GTP, GTP/NaCl and GTP/citrate (**A**) and malate/Pi (**B**) transport activities in liposomes reconstituted with wild-type or ggc1Δ mitochondrial extracts. Transport was started by adding 1 μM [8-^3^H] GTP (**A**) and 0.1 mM [^14^C] malate (**B**) to proteoliposomes preloaded with 5 mM GTP, 5 mM NaCl or 5 mM citrate (**A**) and 20 mM Pi (**B**). The values are the means ± SD of four experiments.

In another set of experiments, we studied the effect of internal citrate on the uptake of GTP into proteoliposomes reconstituted with Triton X-114 extracts of mitochondria isolated from wild-type and *ggc1*Δ yeast strains (Figure 8A). When measured in proteoliposomes reconstituted with wild-type mitochondrial extract, the citrate-induced [8-^3^H]GTP uptake was about 50% of the substantial [8-^3^H]GTP/GTP exchange activity. In comparison, these activities measured in *ggc1*Δ mitochondrial extract proteoliposomes were negligible. As a control, the [^14^C]malate/phosphate exchange (the defining reaction of the dicarboxylate carrier, known as Dic1p [33]) was the same in both wild-type and *ggc1*Δ mitochondrial extract proteoliposomes (Figure 8B), providing evidence that the number of reconstituted extracts was the same. In addition, [8-^3^H]GTP uptake was virtually null when proteoliposomes, reconstituted with wild-type or *ggc1*Δ mitochondrial extracts, contained internally NaCl instead of GTP or citrate (Figure 8A).

### 3.4. Structural Analysis of a Possible Citrate Binding Region on the Ggc1p 3D Model

**Figure 9:**
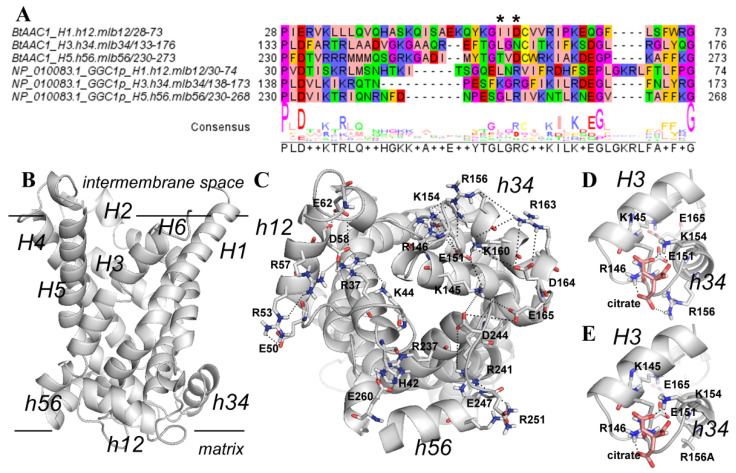
Ggc1p-citrate interactions allowing Ggc1p citrate-mediated uptake of GTP. Panel **A**. Inter-repeat sequence analysis of Ggc1p and BtAAC1. The sequence alignment of the three repeats of Ggc1p and the bovine ADP/ATP carrier (BtAAC1) is reported to highlight residues between the first part and the second part of the MC sequence motif. Amino acids are colored according to the default JalviewZappo style (http://www.jalview.org/, accessed on 15 May 2022). A consensus sequence is reported at the bottom of the figure, where the sequence motif of the mitochondrial carrier family PX[D/E]XX[K/R] EGXXXXAr[R/K]G is brought into evidence. “*” symbols indicate the position of R53, K154, R156, and R251 in the three Ggc1p repeats. Notably, panel (**A**) shows that Ggc1p repeat 2 (the central row of the alignment panel) is the only Ggc1p repeat harboring two basic residues, K154 and R156, which align with the residues of the regulatory motif at the level of the [D/N]C dipeptide observed in the three BtAAC1 repeats, according to [40]. Panel (**B**). The lateral view of the Ggc1p 3D structural model in cytosolic conformation is reported in white cartoon representation. The transmembrane helices *H1-6* and the short helices *h12, h34 and h56* parallel to the membrane plane are indicated by italic black labels. The two horizontal black lines on the top and on the bottom of the Ggc1p 3D model denote the boundaries of the inner mitochondrial membrane. Panel (**C**). The bottom view of Ggc1p is reported in white cartoon representation. Basic and acidic residues participating in the complex ion pair network described in the text are reported in white sticks and labeled. Short helices parallel to the mitochondrial membrane plane are indicated by italics labels (h12, h34, h56). Panels (**D**,**E**). Zoomed views of the proposed citrate binding region in the Ggc1p wild-type model (panel (**D**)) and in the Ggc1p R156A mutant model (panel (**E**)) at the level of residues R156 and K154. Black dashed lines indicate ionic interactions and H-bonds.

Comparative multiple sequence analysis, *in silico* mutagenesis and molecular modeling studies were employed for investigating the role of R53, K154, R156 and R251 in the citrate-induced uniport activity of Ggc1p and for disclosing the binding region of Ggc1p for citrate. The 3D comparative model of the yeast Ggc1p in c-conformation (Figure 9) consists of six helices (H1–6) perpendicular to the membrane plane and three short helices (h12, h34 and h56) parallel to the membrane plane according to the crystallized bovine AAC1, used as a protein template [43], and to other MC 3D comparative models [34,40,50]. A comparative sequence analysis between Ggc1p and the crystallized bovine ADP/ATP carrier brought to our attention the presence of a lysine/arginine enriched region in the yeast Ggc1p (containing residues R53, K154, R156 and R251, Figure 9A), that is aligned with the residues of the three ADP/ATP carrier repeats at the level of the sequence motif 50-Q-Y-K-G-X-X-D-C-X-R-K-60 (ADP/ATP carrier first repeat sequence residues numbering [40]), and could be a candidate binding region for citrate. The four above-mentioned basic residues, R53, K154, R156 and R251, are located in the three short helices parallel to the membrane plane (Figure 9B,C) and appear to be involved in an intricated ion-pair network with close acidic residues of the MC regulatory motif, i.e., E50, E51 and E247, and of the second part of the MC sequence motif, i.e., E62, (D164) E165 and E260 (Figure 9C). In more detail, repeat 1, hosting H1 and H2, shows an ion-pair network involving residues E50 (h12), R53 (h12), R37 (H1), R57 (h12), D58 (h12) and E62 (among the matrix loop residues located in the region known as mlb12 between the helix h12 and H2 [40]), and K44 (among the matrix loop residues located in the region known as mla12 between the H1 and the helix h12 [40]) (Figure 9C). Repeat 3, hosting H5 and H6, shows an ion-pair network involving residues E246 (h56), R251 (h56), R241 (H5), R237 (H5) and E260 (among the matrix loop residues located in the region known as mlb56 between the helix h56 and H6 [40]) and H42 (mla12) (Figure 9C). Notably, repeat 2, hosting H3 and H4 residues, participates in an even more intricated ion-pair network, including residues K145 (H3), K160 (h34), E151 (h34) and D164 (among the matrix loop residues located in the region known as mlb34 between the helix h34 and H4 [40]) as well as E165 (mlb34), D244 (mla56), K154 (h34), R156 (h34), E151 (h34) and R146 (H3) (Figure 9C). Consequently, the replacement of the hindering/positively charged R53, K154, R156 and R251 residues with the shorter/hydrophobic alanine is predicted to weaken the strong H-bond/ionic interaction network observed at the level of the three matrix loops (Figure 9C).

**Figure 10:**
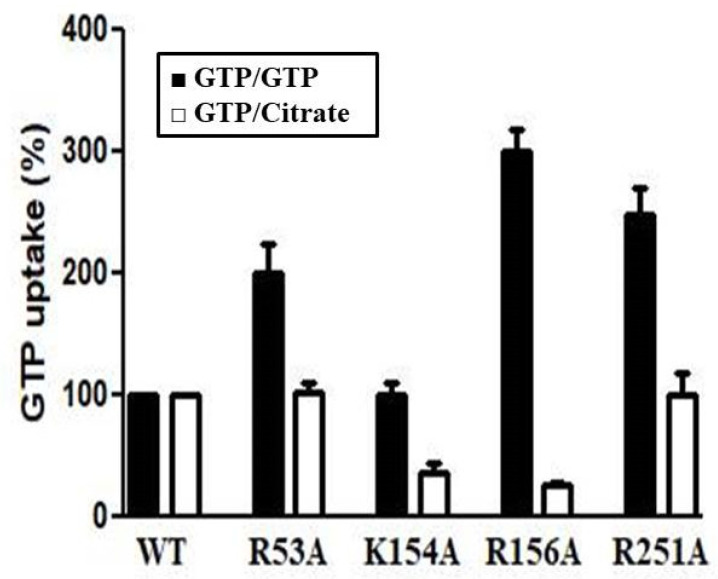
[8-^3^H] GTP uptake in proteoliposomes reconstituted with recombinant wild-type or mutant Ggc1p. Transport was started by adding 1 μM [8-^3^H] GTP to proteoliposomes containing internal 5 mM GTP (antiport, black columns) or 5 mM citrate (CIGU, white columns) and stopped after 20 s. The uptake of WT Ggc1p was considerated 100%.

Transport experiments were performed using proteoliposomes reconstituted with wild-type or mutated Ggc1p (Figure 10). The citrate-induced [8-^3^H]GTP uptake (CIGU) was observed in K154A and R156A mutants but not in R53A and R251A mutants. The fact that CIGU in K154A and R156A mutants were markedly decreased with respect to that of wild-type Ggc1p strongly suggests that K154 and R156 play a crucial role in the binding of citrate to Ggc1p. In contrast, the replacement of R53, R156 or R251 with an alanine, but not of K154, enhanced the [8-^3^H]GTP/GTP exchange activity.

In order to further investigate the Ggc1p region hosting residues K154 and R156, a docking analysis was performed. This analysis revealed 20 poses in similar positions with a root mean square deviation less than 1.0 Å. The 20 generated citrate conformations were grouped by the software in a single cluster of poses ranked according to the free energies of citrate binding. No other cluster of poses was proposed by the software in the investigated binding region. Among the generated conformations, the citrate pose with the lowest predicted free energy is shown in Figure 9D, highlighting a set of stable interactions mainly with K154 (h34), R156 (h34) and, at the same time, with R146 (H3) from the matrix gate area (Figure 9D). Furthermore, Figure 9E shows that the replacement of the hindering/positively charged R156 with the shorter/hydrophobic alanine weakens the citrate binding affinity for the proposed regulatory binding region.

## 4. Discussions

The members of the mitochondrial carrier family catalyze the translocation of solutes across the inner mitochondrial membrane by exchange of substrates (antiport), unidirectional transport of substrates (uniport) or both mechanisms [51,52,53,54]. In a previous study, using proteoliposomes reconstituted with the recombinantly expressed and purified Ggc1p, this MC was shown to catalyze only a counter-substrate exchange [9].

In this work, using the same transport approach (expression-purification-reconstitution assay (EPRA) method [51]), we reinvestigated the specificity of Ggc1p. Quite unexpectedly, it was found that, as with the known substrates of Ggc1p, GTP and GDP, tricarboxylate compounds induce a substantial uptake of externally added labeled GTP when included in the proteoliposomal matrix (Figure 1). Among the various tricarboxylates tested, citrate and 1,2,3-BTA, a known impermeable inhibitor of the mitochondrial citrate carrier [49,55], were the most effective compounds in inducing the transport of GTP into or from proteoliposomes reconstituted with Ggc1p, when present inside or outside the proteoliposomes, respectively (Figure 2, Figure 3 and Figure 4). The possibility that citrate (as the other tricarboxylates) could be transported by Ggc1p, i.e., could be substrates of Ggc1p, was inconsistent with their chemical structure drastically different from that of the GTP and GDP nucleotides and was definitely ruled out by the finding that labeled citrate was not transported by Ggc1p-reconstituted proteoliposomes, regardless of the counter-substrate used (Figure 4). We, therefore, concluded that citrate, when present either inside or outside the proteoliposomes, acts as an effector or regulator of Ggc1p by trans-activating the unidirectional transport of GTP (CIGU) towards the citrate-containing compartment. In other words, Ggc1p, until now known as an obligatory antiporter [9], in the presence of citrate, acquires the ability to function as a uniporter of GTP. This conclusion is substantiated by kinetic evidence (Figure 6) and the fact that citrate does not affect the antiport transport activity of Ggc1p (Figure 7). In addition, the ability of Ggc1p to catalyze CIGU was confirmed using proteoliposomes reconstituted with mitochondrial extracts from the wild-type yeast strain, whereas proteoliposomes reconstituted with mitochondrial extracts from a yeast strain selectively devoided *GGC1* did not show virtually any CIGU (Figure 8).

The data presented in this work also demonstrated that the binding site of Ggc1p for citrate is different from that for the substrate GTP. Thus, (i) the antiport transport activity of Ggc1p is not inhibited by citrate as mentioned above, (ii) the inhibition profile of CIGU is partially distinct from that of the antiport GTP/GTP (Figure 5), and, more importantly, (iii) the mutation of specific basic residues of the binding site of citrate inhibits CIGU, but not the antiport activity of Ggc1p (Figure 10). Furthermore, our data and particularly those obtained from the use of the Ggc1p mutants K154A and R156A and from the docking experiments, give evidence that citrate may bind to the lysine/arginine-rich region located at the level of the matrix loops at the bottom of the Ggc1p 3D model (Figure 9). Interestingly, residues of these loops were recently shown to be involved in interactions with ligands responsible for the modulation of some mitochondrial carrier activity [34,40,56,57,58].

What is the function of the citrate-induced GTP uniport activity of Ggc1p? In order to answer this question, it has to be appreciated that it is generally thought that all mitochondrial carriers are inserted in the inner mitochondrial membrane unidirectionally, i.e., with the N- and C-termini exposed towards the cytosol and the matrix loops towards the matrix [59,60,61,62]. It should also be noted that, because citrate binds to residues of the matrix loops, the “trans” effect of citrate in inducing uniport of GTP, i.e., the transport of GTP towards the citrate-containing compartments (either internally or externally, the Ggc1p-reconstituted proteoliposomes), clearly indicates that the Ggc1p molecules are inserted in the liposomal membrane randomly, i.e., with the N- and C-termini as well as the matrix loops inserted partly outside and partly inside the membrane, as demonstrated for other reconstituted mitochondrial carriers [63,64]. However, it is apparent that the main physiological role of CIGU is to import GTP into the mitochondrial matrix, where this nucleotide is required for the many processes mentioned in the introduction.

**Figure 11:**
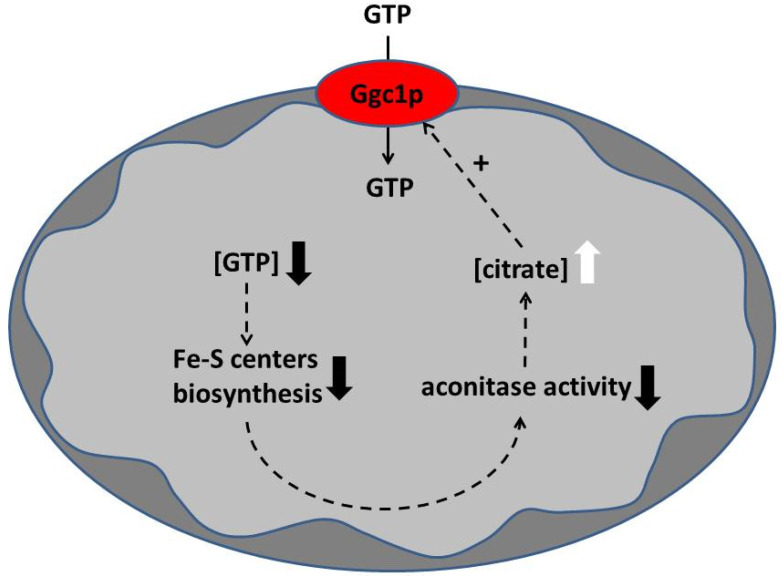
Schematic diagram representing a model linking the level of citrate in the mitochondrial matrix to the GTP level in the same organelles. Squared brackets indicate the concentration of citrate or GTP; downward black arrows indicate a decrease, and upward white arrows indicate an increase. The scheme is depicted to show the stimulating role of citrate in GTP import via Ggc1p (CIGU). However, the same model (with the arrows inverted) can describe the negative effect of an increased intramitochondrial GTP level on the intramitochondrial citrate level leading to reduction in the citrate-induced GTP uptake via Ggc1p (CIGU).

As shown in this work, CIGU is completely dependent on the concentration of citrate (Figure 7) and on the activity of the yeast mitochondrial carrier Ggc1p. In the mitochondrial matrix, citrate is produced by citrate synthase and catabolized by aconitase. Therefore, without considering the amount of citrate transported by the mitochondrial citrate carriers [65,66], the intramitochondrial citrate concentration can be enhanced by an increase in citrate synthase activity or a decrease in aconitase activity. Aconitase is a protein with a [4Fe-4S] center whose synthesis requires GTP. We hypothesize that Ggc1p and aconitase play a key role in controlling the level of GTP in the mitochondrial matrix (Figure 11). 

When the pool of GTP (or more generally of guanine nucleotides) decreases in the matrix, for example, during mitochondrial division, this impacts the synthesis of iron–sulfur centers diminishing the activity of aconitase. The consequent increase in citrate concentration caused by the reduction in the aconitase activity would then activate CIGU, restoring the concentration of GTP in the mitochondrial matrix to standard levels (Figure 11). In line with this proposal, it was shown that GTP in the mitochondrial matrix represents a key element in iron homeostasis [14].

## Data Availability

Not applicable.
